# Baseline Assessment of Taeniasis and Cysticercosis Infections in a High-Priority Region for *Taenia solium* Control in Colombia

**DOI:** 10.3390/pathogens14080755

**Published:** 2025-07-31

**Authors:** Carlos Franco-Muñoz, María Camila Jurado Guacaneme, Sonia Dayanni Castillo Ayala, Sofia Duque-Beltrán, Adriana Arévalo, Marcela Pilar Rojas Díaz, Julián Trujillo Trujillo, Luz Elena Borras Reyes, Luis Reinel Vásquez Arteaga, Julio César Giraldo Forero, Mario J. Olivera

**Affiliations:** 1 Parasitology Group, Public Health Research Division, National Health Institute, Bogotá D.C. 111321, Colombia; mjurado@ins.gov.co (M.C.J.G.); scastillo@ins.gov.co (S.D.C.A.); sofiaduquebeltran@gmail.com (S.D.-B.); aarevalo@ins.gov.co (A.A.); molivera@ins.gov.co (M.J.O.); 2 Emerging, Re-Emerging and Neglected Diseases Group, Sub-Directorate of Communicable Diseases, Ministry of Health and Social Protection, Bogotá D.C. 110311, Colombia; 3 Reference for the Zoonosis and Food Program, Tolima Health Secretary, Government of Tolima, Ibague 730006, Colombia; luzelena.borras@saludtolima.gov.co; 4 Group Center for Studies in Microbiology and Parasitology (CEMPA), Faculty of Health Sciences, Department of Internal Medicine, University of Cauca, Popayán 190003, Colombia; 5 Research Group of the Faculty of Health Sciences (INSAFUSM), San Martin University Foundation of Colombia (Bogotá), Bogotá D.C. 110311, Colombia

**Keywords:** neglected diseases, *Taenia*: One Health, indigenous communities, seroprevalence, KAP survey, parasitic intestinal disease

## Abstract

Coyaima is a town in the department of Tolima, Colombia, that was prioritized in a pilot program under Colombia’s National Plan for the Control of the Taeniasis/Cysticercosis Complex, focusing on this neglected health issue. The project engaged local indigenous communities, promoting education and outreach within the One Health framework. The study included 444 randomly selected volunteers, who filled a Knowledge, Attitudes, and Practices (KAP) survey on the taeniasis/cysticercosis complex. The baseline study found no *Taenia* spp. eggs via microscopy on 383 stool samples examined, and no *T. solium* DNA was detected on human stool and soil samples by Copro-qPCR. However, seroprevalence was 8.5% for human cysticercosis and 14% for porcine cysticercosis, as detected by in-house ELISA testing for *T. solium*. Moreover, 57.9% of participants who provided a stool sample were positive for at least one parasite. Following the sampling and characterization activities, local health workers implemented mass treatment with Niclosamide, based on evidence of ongoing transmission, high porcine seroprevalence, poor basic sanitation, and the presence of free-roaming pigs reported in the KAP survey. These findings provide scientific evidence to apply national public health policies for controlling taeniasis/cysticercosis complex in Coyaima.

## 1. Introduction

Human intestinal infection by the tapeworm *Taenia solium*, known as taeniasis, and cysticercosis—a disease caused by the accidental ingestion of parasite eggs of the *T. solium* developing into larvae in various human tissues, including the brain (neurocysticercosis, NCC)—constitute a major interconnected public health challenge, particularly in regions where socioeconomic vulnerability and limited access to healthcare facilitate transmission [1,2,3,4]. The World Health Organization’s Foodborne Disease Burden Epidemiology Reference Group has identified *T. solium* as a leading cause of mortality from foodborne disease [1]. Moreover, the social and economic burden of these diseases is substantial, adversely affecting quality of life, driving up healthcare expenditures, and impairing workforce productivity [5,6]. Additionally, *T. solium* infects pigs, leading to porcine cysticercosis [7].

The life cycle of *T. solium* is complex, involving humans as the only definitive host and pigs as the intermediate host [1,8]. Humans acquire taeniasis by consuming undercooked or raw pork contaminated with cysticerci, which matures into adult tapeworms within the intestine over a period of 3–5 months. The gravid proglottids are then released and excreted in feces, containing microscopic, fertilized eggs of *T. solium*, which contaminate the environment and serve as a source of both human and porcine cysticercosis. For this reason people who do not eat pork can acquire cysticercosis either exogenously via fecal–oral contamination or endogenous autoinfection due to reverse peristalsis [8,9,10,11,12] and free-roaming pigs are in high risk of acquiring cysticercosis due to their coprophagial behavior or eating contaminated food/water [13,14]. Despite their role in perpetuating the transmission cycle, human carriers of adult tapeworms are frequently asymptomatic, although some may present mild, non-specific symptoms such as abdominal pain, anorexia, weight loss, and digestive discomfort [15,16].

In cases of NCC, clinical manifestations primarily include severe neurological symptoms such as seizures or epilepsy (78.8%), chronic headaches (37.9%), focal deficits (16.0%), and signs of increased intracranial pressure (11.7%). In severe cases, NCC can be fatal. It is well established that NCC accounts for up to 30% of acquired epilepsy cases in endemic regions [9,11,17,18,19].

Taeniasis and cysticercosis are endemic to sub-Saharan Africa, Latin America, and regions of East, South, and Southeast Asia. According to the World Health Organization (WHO), 51 out of 196 assessed countries are classified as endemic. However, subnational distribution remains poorly delineated, posing a barrier to targeted intervention [20].

Rodriguez-Rivas et al. [21] analyzed hospitalization rates for NCC in Colombia from 2009 to 2019, reporting an overall increase. The lowest rate was recorded in 2013 (0.49 per 100,000 inhabitants), while the highest was in 2019 (2.33 per 100,000), based on data from the Individual Health Records System (“Registro Individual de Prestación de Servicios”, RIPS).

More recenty, Rosselli y Pantoja-Ruiz [22] reported a prevalence of 7.1 cases of NCC per 100,000 inhabitants using RIPS data from 2017 to 2021. However, these figures account only for NCC cases that received specialized medical care, likely underestimating the true burden of disease due to asymptomatic or undiagnosed cases.

The control of the taeniasis/cysticercosis complex was declared a public health priority by the Colombian Ministry of Health and Social Protection in 2017. To address this, the National Technical Board—a cross-sector committee—was established to develop and implement the “National Guidelines for Control the Taeniasis/Cysticercosis Complex” [23]. As part of this strategy, piloting elimination programs in at least one selected municipality was deemed necessary to gather comprehensive technical and scientific data.

In this context, historical records of porcine cysticercosis led to the identification of Coyaima, Tolima, as a priority area. Serrano et al. [24] reported a seropositivity rate of 25.64% (30/117) in this region, while Giraldo Forero et al. [25] found a *Taenia* spp. carrier prevalence of 2% (3/159) and a porcine cysticercosis seroprevalence of 17% (17/102).

This study aimed to establish the baseline coproprevalence of taeniasis and seroprevalence of human and porcine cysticercosis in Coyaima, a municipality characterized by risk factors such as free-ranging pig farming, inadequate sanitation, and open defecation—highlighting an ongoing public health concern. Given that the coproscopic techniques also allow us to find parasites other than *Taenia* spp., this study also aimed to make visible other parasitic infections that affect the population’s health.

## 2. Materials and Methods

### 2.1. Study Area

Coyaima is a town in the department of Tolima, Colombia (3°47′51″ N, 75°11′38″ W). It is in the Andean region at an altitude of 392 m above sea level, with an average temperature of 26 °C. The municipality comprises 6.20 km^2^ of urban area (1% of the total) and 658.13 km^2^ of rural area (99%), inhabited by 18.9% and 81.1% of the population, respectively, live distributed across 54 microterritories.

According to the National Administrative Department of Statistics (DANE, in Spanish), the adjusted post-COVID-19 population in 2024 was 23,363. The majority of Coyaima’s population (84%) identifies as indigenous. This study was formally approved in writing by indigenous organization leaders, including the Regional Indigenous Council of Tolima (CRIT), the Federation of Indigenous Autonomous Councils of Tolima (FICAT), and the Association of Indigenous Councils of Tolima (ACIT). Additionally, the local contracted personnel have the recognition and endorsement of indigenous communities within the territory.

### 2.2. Methodological Design

A Simple Random Sampling (SRS) approach was used to establish the baseline prevalence of the taeniasis/cysticercosis complex, based on a sampling frame of 7602 families provided by the local health department. An assumed prevalence of 50% was applied—despite known rates of 1–2%—to ensure a conservative, maximum sample size. The sample was calculated with a 95% confidence level and a 5% margin of error using EpiInfo software (Version 7.2.4.0) (Centers for Disease Control and Prevention, CDC) in the StatCalc module. Additionally, a 20% adjustment was included to account for participant dropout.

Household randomization was performed proportionally across the 54 microterritories using the Microsoft Excel = RANDBETWEEN() function, and results were cross-referenced with household registries by local leaders and authorities.

The sampling strategy involved at least two household visits, organized geographically. During the first visit, participants were informed about the study’s objectives and intervention plan. If a household declined participation or was unavailable, it was replaced by the next household on the list or by selecting a new random number. Household was used as secondary sampling unit (SSU), but only one person was chosen for the study, preferably the person who prepared the food, as a representative of the health of their family nucleus. Informed consent was obtained before administering the KAP survey on the taeniasis/cysticercosis complex.

The second visit involved biological sample collection:

Human samples: Stool and blood samples were obtained from participating individuals.

Porcine samples: Backyard pig owners were surveyed, pigs underwent sublingual examination, and porcine blood samples were collected with owner’s written consent, provided sampling was feasible.

Environmental samples: Soil samples were collected from households where open defecation was reported.

### 2.3. Knowledge, Attitudes, and Practices (KAP) Survey on the Taeniasis/Cysticercosis Complex

The KAP survey was developed based on prior surveys from the University of Antioquia [26], the National University of Colombia, and the Research Group of the Cayetano Heredia University of Peru (Global Health Center of Tumbes). Additionally, the National Technical Board of the Taeniasis/Cysticercosis Complex of Colombia reviewed and contributed to its development.

To ensure linguistic and contextual appropriateness, the survey terminology was adjusted to align with the common language used in Coyaima. This adaptation was validated through a participatory exercise involving local teachers and community members.

The final survey consisted of 41 questions, categorized into four sections: Sociodemographic Data; Human Health Information; Pig Health and Husbandry; and Environmental Factors.

A Spanish-language instruction manual for field staff was also developed. The survey was administered in a physical format, and responses were subsequently digitized and coded in Microsoft Excel (Supplementary Table S1).

### 2.4. Sample Collection and Preservation

Following the One Health approach, human feces and blood, pig blood, and soil samples were collected from the 54 microterritories in Coyaima. Blood samples were transported to the local hospital (E.S.E. Hospital Municipal San Roque), where the serum was stored at –20 °C for further analysis. Fecal and soil samples were preserved using two different solutions: SAF (0.2 M Sodium Acetate, 2% Acetic Acid, and 5% Formalin) to maintain parasite morphology, and 100% molecular-grade ethanol for molecular analysis. Samples were periodically sent to the Parasitology Laboratory of the National Health Institute (NHI) in Bogotá, D.C. The selected preservations ensure accurate microscopic and molecular diagnosis, even when stored at room temperature [27,28].

### 2.5. Microscopic Parasites Identification

Coproparasitological analysis was performed at the NHI-Parasitology Laboratory using the Kato–Katz and modified formalin–ether (SAF–ether) microscopic techniques. The collected samples were filtered, and 10 mL of the SAF-diluted sample was used for each technique.

For the SAF–ether method, 1 mL of ether was added to the fecal samples, mixed by inversion, and centrifuged at 500× *g* for 10 min. The upper three layers of the supernatant were discarded, leaving only the precipitate for microscopic examination. A drop of saline solution and a drop of Lugol’s iodine were placed on opposite sides of a microscope slide, then mixed with a drop of the sample and analyzed microscopically to identify and classify parasitic forms in the fecal and soil samples.

For the Kato–Katz technique, the filtered fecal and soil samples were diluted 1:3 with SAF solution and centrifuged at 500× *g* for 10 min. A fecal matter pellet of 41.7 mg was formed using a plastic template (6 mm diameter, 1.5 mm thick). A piece of methylene blue-dyed cellophane, pre-soaked in a glycerin and water solution, was used to cover the pellet. The sample was spread across the cellophane using an applicator and then examined under a microscope. Each sample was mounted in duplicate [29,30].

### 2.6. Molecular Analysis

DNA extraction from fecal and soil samples was conducted following the protocol described by Franco-Muñoz et al. [31]. Samples preserved in 100% ethanol were filtered and centrifuged at 16,000× *g* for 5 min. The resulting pellet was reconstituted in 200 µL of 70% molecular-grade ethanol to hydrate the DNA, then dried at room temperature; samples with at least ≥20 mg were used for individual DNA extraction, and samples with >50 mg were pooled (~50 mg each) leaving enough sample for future confirmations.

The samples underwent a pretreatment incubation with 5 M EDTA solution (pH 8.0) at 55 °C and 1500 rpm for 1 h to inhibit DNases. After centrifugation, the pellet was lysed using ASL buffer (Qiagen, Hilden, Germany) and Proteinase K (Roche, Basel, Switzerland), incubated at 55 °C and 1500 rpm for 2 h. DNA extraction was then performed following the QIAamp DNA Stool Mini Kit (Qiagen, Hilden, Germany) manufacturer’s protocol.

For soil samples, dried samples were processed in pairs, with 50–100 mg of each sample used for extraction, following the Nucleospin Soil Kit (MACHEREY-NAGEL, Düren, Germany) manufacturer’s protocol.

The quantity and quality of the extracted DNA were assessed using a Nanodrop 2000 spectrophotometer (Thermo Scientific, Waltham, MA, USA). A multiplex qPCR assay was conducted to specifically detect a fragment of the ITS gene of *Taenia solium* and *Taenia saginata*, using primers reported by Praet et al. [32]. Amplification of an 18 S mammalian gene fragment [33] served as an endogenous control for extraction and amplification (Table 1).

The qPCR was performed using 2 µL of DNA, 1 X Luna^®^ Universal Probe qPCR Master Mix (NEB, Ipswich, MA, USA), 0.1 µM of each primer, and 0.0625 µM of each probe, in a final volume of 15 µL. A CFX96 Touch Real-Time PCR Detection System (Bio-Rad, Hercules, CA, USA) was used with the following thermal profile: initial denaturation at 95 °C for 5 min, followed by 45 amplification cycles of 95 °C for 15 s and 60 °C for 31 s. Each sample was processed in duplicate, and results were analyzed using CFX Maestro Software 3.1.

### 2.7. Serological Test

The Indirect Enzyme-Linked Immunosorbent Assay (ELISA) was performed at the Group in Parasitology and Tropical Microbiology (GIPAMT) in Bogotá, following the standardized protocol established by Giraldo Forero et al. [25]. This ELISA utilized the 53 kDa antigenic fraction of *T. solium* isolated by the authors at a concentration of 0.4 µg using a 96-well Clear Flat Bottom Polystyrene TC-treated Microplate (Corning Ref. 3595). Each plate included a positive control (confirmed by microscopy) and a negative control of serums available from endemics areas. These samples were confirmed for other parasite infections (protozoan, helminths, and cestode) using coproparasitologic techniques. Additionally, *T. saginata* serum from Cauca was confirmed by the copro-qPCR described previously for human assays. For porcine experiments, positive serums from pigs, confirmed by live and postmortem examination of cysticercosis, were used and serums confirmed by stool examination for other parasite infections (protozoan and helminths)

Briefly, human and porcine serum samples were diluted 1:100 in phosphate-buffered saline (PBS) and incubated overnight at 4 °C with the antigen. The samples were then washed with PBS-Tween 20 (0.1%), and the wells were blocked with 0.1% bovine serum albumin (fraction V, SIGMA, St. Louis, MO, USA). The reaction was developed using a peroxidase-conjugated secondary antibody—anti-human or anti-pig IgG—at dilutions of 1:2500 and 1:5000, respectively. Absorbance values were measured at 492 nm using a Multi-Skan Plus MK-II ELISA reader.

To establish the cut-off value, the mean absorbance of the negative control was calculated, and two standard deviations were added.

## 3. Results

### 3.1. Parasite Identification

**Figure 1:**
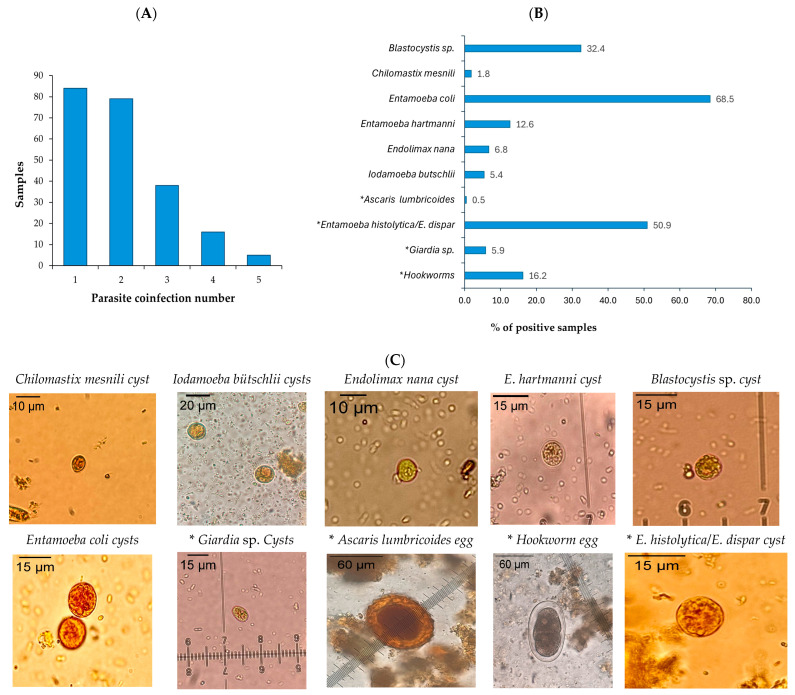
Microscopic parasite identification. (**A**) Samples with multiple parasites identified through coproparasitological microscopy. (**B**) Parasitic entities detected in stool samples using coproparasitological methods. (**C**) Photographs of cysts and eggs with magnification objective of parasite entities of stool samples preserved with SAF visualized in montage with Lugol’s iodine staining. Image J (v. 1.54 g) was used to calibrate the photographs. * Pathogenic parasites are marked with an asterisk.

Of the 444 participants, 383 stool samples were obtained for microscopic analysis, revealing a high prevalence of intestinal parasites. A total of 57.9% (222/383) samples contained at least one parasite species, while 36% showed polyparasitism, with individuals harboring two and five different species (Figure 1A). The protozoan pathogens detected included the *Entamoeba histolytica*/*E. dispar* complex (113 samples) and *Giardia* sp. (13 samples). Despite multiple tests (up to four times per sample), no eggs of *Taenia* spp. were detected.

Additional commensal or non-pathogenic protozoa identified included *Entamoeba coli*, *Entamoeba hartmanni*, *Endolimax nana*, *Iodamoeba butschlii*, *Chilomastix mesnili*, and *Blastocystis* sp. (Figure 1B).

Among the 125 soil samples processed, 11.2% (14/125) tested positive for structures compatible with parasite eggs and larvae (such as *Trichuris trichiura* and *hookworms*) under microscopic analysis. Given the limitations of microscopy for *T. solium* identification, a molecular analysis was conducted to detect *T. Solium* DNA of 376 of the 383 fecal samples (seven samples were excluded due to insufficient sample) from Coyaima and all the soil samples. After DNA extraction, a multiplex qPCR for *Taenia* spp. was performed, successfully identifying *T. saginata* and *T. solium* controls. However, none of the field-collected fecal or soil samples tested positive for either species, as confirmed by amplification of the internal control (16 S) in 100% stool samples and 90.4% (113/125) soil samples. These results align with the microscopy findings.

### 3.2. Seropositivity to Cysticerci in Humans and Pigs

**Figure 2:**
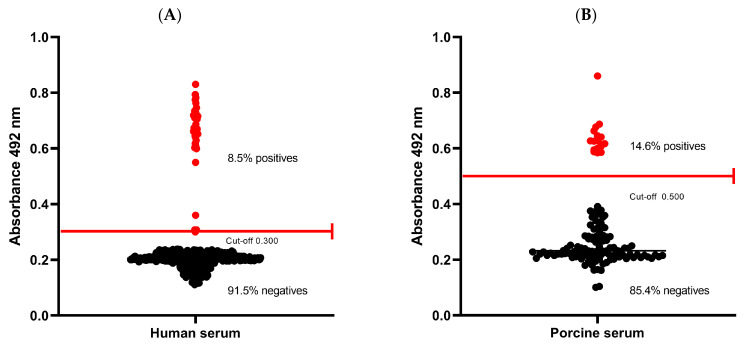
Seropositivity to cysticerci in humans and pigs. (**A**) Human serum. (**B**) Porcine serum. (Red lines represent the ELISA cut-off).

To establish the baseline seroprevalence of cysticercosis in Coyaima, an ELISA test was performed with 435 serum samples of the 444 participants (nine participants did not agree to the blood test or it was not possible to take the sample) to detect antibodies against a native *T. solium* cysticercus antigen. Human seropositivity was 8.5% (37/435) (Figure 2A), indicating prior exposure to *T. solium* in the population. This finding aligns with Coyaima’s classification as an endemic area for the disease.

In pigs, seropositivity was higher at 14.6% (17/116) (Figure 2B), suggesting significant environmental exposure to the parasite. Notably, the sampled pigs were mostly purchased (78%) and were of six months old on average, suggesting recent exposure and raising concerns about potential ongoing transmission in the region (data on pigs is summarized in Supplementary Table S2). These results highlight the need for continued monitoring as well as enhanced control and prevention strategies to mitigate the risk of infection.

### 3.3. KAP Survey

Of the 440/444 participants who completed the KAP survey on the taeniasis/cysticercosis complex (four surveys were excluded of the analysis), 71% (314/440) were women, 95% (418/440) lived in rural areas, and 98% (432/440) self-identified as indigenous (Table 2). Regarding awareness, only 12% (53/440) recognized the disease caused by tapeworms, and 9% (38/440) had heard of human cysticercosis. In addition, 26% (113/440) had heard of the parasite affecting pigs, but only 46% (52/113) understood its transmission mechanism. Just 23% (102/440) reported purchasing pork from authorized vendors, and 14% (60/440) admitted consuming pork containing larval cysts—referred to locally as “pepa” a colloquial term for the cysticerci of *T. solium* found in infected meat.

Among the surveyed population, 43% (189/440) raised pigs, of whom 27% (51/189) allowed pigs to roam freely, 9% (17/189) had pigs with access to human feces, 64% (120/189) slaughtered pigs on their farms, and 12% (22/189) stated they would consume or sell infected pigs, indicating a lack of awareness about the associated health risks.

Concerning basic sanitation conditions, 71% (312/440) lacked access to potable water, 36% (158/440) practiced open defecation, and 39% (170/440) cultivated crops, of which 75% (128/170) irrigated them with river, stream, or well water—an approach that increases contamination risks given the prevalence of open defecation in the community.

## 4. Discussion

### 4.1. Coyaima

This study aimed to establish the current transmission status of the taeniasis/cysticercosis complex (TCT) caused by *T. solium* in a rural Colombian town that has been previously reported as affected by these diseases [24,25]. Applying the One Health approach to TCT control, as recommended by the WHO [34], and recognizing that the parasite’s life cycle involves humans, pigs, soil, water, and food within predominantly indigenous community, this study and intervention was carried out by two human health professionals and one animal health professional in the field. Their work was further enriched by the involvement of a professional anthropologist for several weeks, ensuring that the intervention took into account the community’s worldview.

**Figure 3:**
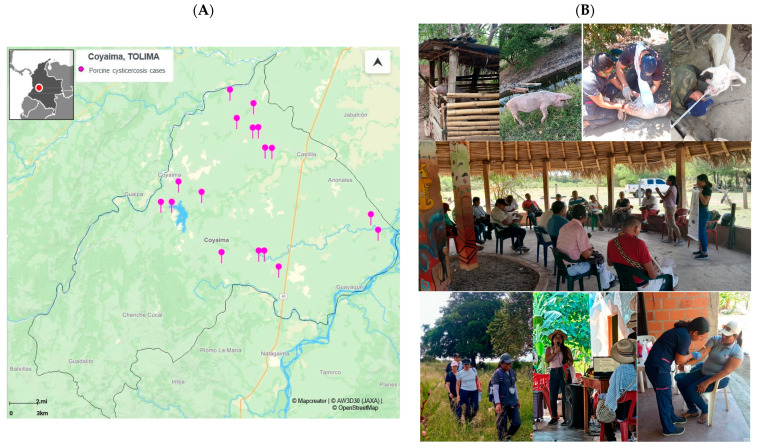
Seropositivity distribution of porcine cysticercosis and photographs of the intervention in Coyaima. (**A**) Map of seropositive porcine samples for cysticercosis cases (https://online.mapcreator.io/ (accessed on 20 March 2025)). (**B**) Photographs of the intervention activities within the Coyaima community and pigs, taken by field staff.

Seropositivity to porcine cysticercis of 14.6% was found (Figure 3A), suggesting possible ongoing transmission of the parasite in Coyaima, Tolima, supported by the young pig age (average of 6.23%), the persistence of risk factors, including free-ranging pig farming, inadequate sanitation, and open defecation, highlighting the need for targeted intervention strategies as the actual intervention performed in this project (Figure 3B).

Coyaima’s economy is primarily based on agriculture, handicrafts, and raising livestock such as pigs and sheep. In 2022, 78.6% of pigs were raised in backyard systems, 21.3% for family or commercial use, and none in industrial settings. By 2018, water and sewer coverage reached only 35.5% and 19.1%, respectively. Health insurance was subsidized for 94.4% of residents, with only 5.6% in the contributory system [35,36,37]. According to the results of the present study, this situation has not changed in recent years; therefore, risk factors persist that could explain the transmission of the disease in this municipality.

The results of the KAP survey revealed limited awareness and widespread risk behaviors related to *T. solium*. While 12% of participants were familiar with taeniasis (commonly known as tapeworm infection, “solitaria” (loner) in Spanish), 9% had heard of human cysticercosis, and 26% had some knowledge of the pig parasite. However, only 46% of those who had heard of it understood its transmission mechanism. These findings indicate that porcine cysticercosis is more widely recognized than human taeniasis, aligning with previous studies that associate *Taenia* primarily with pig diseases [38].

According to our survey, pig farming is common (43% of households surveyed raise pigs), but certain practices increase the risk of infection; 27% of pig farmers allow their animals to roam freely, while only 9% reported that their pigs have access to human feces. While 69% of the population consume pork, only 23% purchase it from authorized sellers, and 14% admitted consuming pork containing cysticerci. Similar behaviors have been reported in other studies in Colombia, where 20.9% (40/192) of surveyed individuals acknowledged eating visibly infected meat [26]. Some even preferred it, describing the texture as “juicy” and consuming it with lemon juice or “caña agria” (*Costus spicatus*). These practices raise serious public health concerns, as they reflect a widespread lack of awareness regarding the associated risks.

Sanitation conditions in Coyaima remain precarious, with 71% of respondents lacking access to potable water. Drinking water is sourced from multiple origins, mainly wells with pumps and community aqueducts. Practicing handwashing after handling garbage or animals is as a protective factor against seropositivity for *T. solium*, and access to safe water was identified an important strategy for the control of TCT and other health problems.

Wastewater disposal is also inadequate, with 93.1% of households discharging it into open fields. The most common sanitation facilities include open defecation areas, standard flush toilets, and rural latrines. These conditions facilitate the persistence of the *T. solium* life cycle, which correlates with the high prevalence of intestinal parasites found in this study. These findings, along with previous reports, underscore the urgent need for comprehensive and sustainable interventions aimed at improving health, basic sanitation, and community education (Figure 3B). Such measures are essential to reducing the transmission of *T. solium* and other parasites in this and other vulnerable communities with similar conditions [2,39,40].

### 4.2. Microscopy

Stool sample analysis using microscopic methods (coproscopy) continues to be the most used approach for detecting and identifying *Taenia* spp. eggs or proglottids. Procedures such as direct smear, Kato–Katz, and formol–ether concentration are routinely applied for this purpose. Nonetheless, these diagnostic tools exhibit limited sensitivity due to the sporadic release of eggs and gravid proglottids in feces [41]. A meticulous microscopic examination of fecal samples was conducted using the SAF–ether and Kato–Katz techniques to identify parasites. The formol–ether technique, modified with SAF solution, relies on density and solubility principles to concentrate parasites in fecal samples while preserving them for microscopic analysis [42,43]. The Kato–Katz technique is widely recommended for detecting and quantifying helminth eggs in feces, particularly in epidemiological research and parasitic disease monitoring. Its simplicity and effectiveness make it a valuable tool for assessing helminth burden and evaluating the impact of control interventions [29].

In this study, no *Taenia* spp. eggs or proglottids were identified using the SAF–ether and double Kato–Katz techniques.

The analysis of 383 fecal samples revealed that 58% contained at least one type of intestinal parasite. Some cases involved polyparasitism with up to five different parasite species identified in 6% of the samples. These findings highlight the presence of fecal–oral transmission routes and poor hygiene practices affecting more than half of the population. A comparison with the study by Gastiaburu [44], which focused on indigenous children and young people in the Chenche Zaragoza microterritory of Coyaima, showed similar proportions of positive samples (52.9%), with *E. Histolytica*/*E. dispar* (25.0%), *Blastocystis* sp. (23.4%), and *E. coli* (14.7%). Unlike previous studies, which focused on children, this study primarily assessed adults (over 90% of participants). Despite the age difference, the consistent patterns of parasitic infection emphasize the ongoing issue of fecal–oral contamination in the region.

The detection of pathogenic protozoa such as the *E. histolytica*/*E. dispar* complex in 113 samples and *Giardia* sp. in 13 samples underscores serious public health concern, particularly for children, who are more vulnerable to these parasites. *E. histolytica* is well known for its pathogenic potential, causing both intestinal and extraintestinal amebiasis, including hepatic amebiasis [45]. Meanwhile, *Giardia* sp. is responsible for giardiasis, a gastrointestinal disease transmitted through contaminated water or food, which can lead to malabsorption issues and stunted growth in children [46,47].

Additionally, other protozoa, including *E. coli*, *E. hartmanni*, *Endolimax nana*, *Iodamoeba butschlii*, *Chilomastix mesnili*, and *Blastocystis* sp., were identified. These protozoa are described as non-pathogenic, but they indicate fecal–oral transmission, and hence, they show the presence of risky practices, indicating a wide range of infectious agents within the community. Although *E. coli* is generally considered a non-pathogenic intestinal commensal [48,49], its presence in large numbers has been associated with gastritis, indigestion, and other gastrointestinal issues [50].

The detection of helminths such as *Hookworms* and *Ascaris lumbricoides* further highlights the need for effective control and prevention measures. Uncinariasis and ascariasis can lead to anemia, malnutrition, and other severe complications [51,52,53,54]. These findings align with previous studies in the norther Andean region of Colombia, indicating a persistent vulnerability to these infections. *Hookworm* (uncinariasis) infection is a common disease in tropical and subtropical regions with poor sanitation, where contact with contaminated soil is frequent. Transmission typically occurs through the skin, making individuals who work in agriculture or have regular contact with the soil, such as those in the population studied who had agriculture activities, particularly vulnerable to infection. Early detection and treatment, particularly in children, is crucial to preventing nutritional deficiencies, growth delays, and other long-term health impacts, ensuring healthy development during early childhood [51,55,56].

### 4.3. Molecular Analysis

The extraction of DNA from fecal and soil samples preserved in absolute ethanol was successful for this sample type. However, multiplex qPCR for *Taenia* spp. did not detect *T. solium* DNA in the analyses, which aligns with the microscopy results, despite other studies reporting greater sensitivity of qPCR compared to the Kato–Katz technique [57,58]. On the other hand, the endogenous extraction control (16S) was successfully amplified in 100% of fecal samples and 93% of soil samples, confirming that the samples were properly processed and that the absence of *T. solium* detection was not due to test inhibition [59]. Real-time PCR results provided evidence of amplification of the endogenous control, and the *T. solium* and *T. saginata* controls in stool and soil samples are provided in Supplementary Figure S1 and Supplementary Data.

In soil samples, parasite-like structures were observed in 11.2% of cases, including eggs and larvae, which correlates with the fact that 36% of participants reported practicing open defecation.

The analysis of samples from the Coyaima community using the One Health approach and various parasitological and molecular techniques did not detect *Taenia* spp. eggs, despite the region being endemic to taeniasis. This highlights the low sensitivity of conventional microscopy methods, which rarely detect a prevalence above 3% in endemic areas [60]. The intermittent excretion of *Taenia* eggs contributes to false negatives, underscoring the need for more sensitive diagnostic methods that do not rely on the presence of eggs or proglottids, as current techniques tend to underestimate taeniasis prevalence [61].

### 4.4. Serology

The detection of anti-*T. solium* antibodies using the ELISA technique in 8.5% (37/435) of the human population and 14% (17/121) of the pigs evaluated suggests that they have been exposed to *T. solium* at some point, either to the cysticercus stage or the adult form. Since humans may have acquired the infection elsewhere, the presence of porcine cysticercosis in Coyaima pigs—whose average lifespan is 6.3 months (ranging from 2 to 18 months)—suggest recent exposure and possible ongoing *Taenia* transmission within the community. Given the relatively short lifespan of these animals (typically about one year) and the limited movement of free-ranging pigs, which usually travel between 50 and 100 m and up to 3 km from their home in search of food and water (where they may consume human feces), they serve as real-time indicators of ongoing local transmission [62,63].

The geographic distribution of seropositive pigs suggests the presence of potential transmission hotspots in specific microterritories in the north, center, and east of Coyaima (Figure 3A). Considering that in these regions, both pigs and humans are exposed to common sources of parasitic infection, it was found that the south region is less populated and focused on agriculture. Identifying this shared transmission zone is crucial for targeting public health interventions guiding the second or third round of the mass drug administration (MDA) and focusing the efforts on implementing control and prevention measures specifically in this area to reduce the spread of parasites (Figure 3A). These findings highlight the importance of an integrated and coordinated public health approach, considering the interconnection between human—animal—ambiental health in the One Health approach [34,64,65], as we see in Figure 3B, considering the worldview and culture of this indigenous community.

Compared to the study by Giraldo et al., which used the same ELISA technique in four microterritories in Coyaima [25], the seroprevalence of porcine cysticercosis in this study is similar (17%, 17/102) to that reported for the period of 2012–2013 but significantly lower than the prevalence reported by Serrano et al. [24], suggesting an improvement in the conditions. Additionally, Agudelo Flórez et al. reported a seroprevalence of 8.7% in humans and 20.9% in pigs in a municipality in northwestern Colombia using the immunoblot technique, aligning with this study [66]. In another municipality in northern Colombia, a recent study reported a porcine cysticercosis seroprevalence of 9.7% (46/472) using a commercial kit [26], which is lower than the prevalence reported in this study.

## 5. Conclusions

The combined analysis of soil samples, human fecal samples, and positive serological tests in both humans and pigs suggests continued active transmission of *T. solium* in Coyaima, a region with poor sanitary conditions that includes backyard pig farming, which explains the persistence of the parasite cycle. Although our tests did not detect *T. solium* DNA or *Taenia* spp. eggs, we observed a high prevalence of other intestinal parasitic infections of public health concern and the importance of continuing to implement interventions with an integrated One Health approach and further evaluation of their impact.

## Figures and Tables

**Table 1:** Primers and probes for the detection for Multiplex qPCR *Taenia* spp.

	Target Species	Oligonucleotide Sequence 5′–3′	Source
*Taenia saginata*	Tsag_ITS_F529	GCGTCGTCTTTGCGTTACAC	[32]
	Tsag_ITS_R607	TGACACAACCGCGCTCTG	
	Tsag_ITS_581 Tq	CCACAGCACCAGCGACAGCAGCAA	
*Taenia solium*	Tsol_ITS_145 F	ATGGATCAATCTGGGTGGAGTT	
	Tsol_ITS_230 R	ATCGCAGGGTAAGAAAAGAAGGT	
	Tsol_ITS_169 Tq-	TGGTACTCTGCTGTGGCGGCGG	
*Mammal*	MammalF	CGACCTCGATGTTGGATCAG	[33]
	MammalR	GAACTCAGATCACGTAGGACTTT	
	MammalP	CCCGATGGTGCAGCCGCTATTAAA	

**Table 2:** Sociodemographic data from KAP survey.

Humans KAP Survey n = 440 Age (Average) = 51.8 Years			
Characteristics		Number	Percent
Sex	Male	128	29%
	Female	312	71%
Resident	Urban	11	3%
	Rural	419	95%
	Both	10	2%
Ethnicity	None	7	2%
	Indigenous	431	98%
	Other	2	0%
Educational level	None	51	12%
	Incomplete primary education	174	40%
	Completed primary education	71	16%
	Incomplete secondary education	36	8%
	Completed secondary education	69	16%
	Incomplete technical education	3	1%
	Completed technical education	10	2%
	Incomplete professional education	1	0%
	Completed professional education	16	4%
	No response	9	2%

## Data Availability

The original contributions presented in this study are included in the article/Supplementary Materials. Further inquiries can be directed to the corresponding author.

## Supplementary Materials

The following supporting information can be downloaded at: https://www.mdpi.com/article/10.3390/pathogens14080755/s1, Supplementary Table S1: KAP results; Supplementary Table S2: Pig’s epidemiological data; Supplementary Figure S1: Real time PCR results; Supplementary Data: Real time PCR results.
