# Supplementary material for: Baseline Assessment of Taeniasis and Cysticercosis Infections in a High-Priority Region for Taenia solium Control in Colombia
Source: Pathogens. 2025 Jul 31;14(8):755. doi: 10.3390/pathogens14080755 (PMC12388895; doi:10.3390/pathogens14080755)
Supplement: Supplementary file 1 [file pathogens-14-00755-s001.zip › Supplementary data. Real time PCR results.pdf]

Ensayo #20 SC 72-93 Pool MF 49-56, 249, 251, 259, 264 2024-02-06 12-44-56_CT044639.pcrd

06/04/2025 14:38

Report Information

User: BioRad/admin
Data File Name: Ensayo #20 SC 72-93 Pool MF 49-56, 249, 251, 259, 264 2024-02-06 12-44-56_CT044639.pcrd
Data File Path: C:\Users\LABORATORIO\Desktop\Taenia spp
Well Group Name: All Wells
Report Differs from Last Save: Yes

Run Setup

Run Information

Run Date: 02/06/2024 12:46
Run User: admin
Run Type: User-defined
Plate File: Placa qPCR Taenia spp Ensayo #20 06-02-2024 .pltd
ID:
Notes:
Sample Volume: 15
Temperature Control Mode: Calculated
Lid Temperature: 105
Base Serial Number: CT044639
Optical Head Serial Number: 785BR23462

Protocol

1: 95.0°C for 5:00
2: 95.0°C for 0:15
3: 60.0°C for 0:30
Plate Read
4: GOTO 2, 45 more times

Plate Display

	1	2	3	4	5	6	7	8	9	10	11	12
A	Pos-1<br><br>ITS TSAG<br>16S MAMMAL<br>POS CONT T sag	Unk-3<br>ITS T SOL<br>ITS T SAG<br>16S MAMMAL<br>Soil 72 & 73	Unk-7<br>ITS T SOL<br>ITS T SAG<br>16S MAMMAL<br>Soil 81 & 82	Unk-11<br>ITS T SOL<br>ITS T SAG<br>16S MAMMAL<br>Soil 89 & 90	Unk-15<br>ITS T SOL<br>ITS T SAG<br>16S MAMMAL<br>STOOL POOL 49	Unk-19<br>ITS T SOL<br>ITS T SAG<br>16S MAMMAL<br>Stool POOL 53	Unk-23<br>ITS T SOL<br><br>16S MAMMAL<br>Stool sample249			NTC<br>ITS T SOL<br>ITS T SAG<br>16S MAMMAL<br>NTC CLEAN ROOM	NTC<br>ITS T SOL<br>ITS T SAG<br>16S MAMMAL<br>NTC ADN ROOM	
B	Pos-1<br><br>ITS TSAG<br>16S MAMMAL<br>POS CONT T sag	Unk-3<br>ITS T SOL<br>ITS T SAG<br>16S MAMMAL<br>Soil 72 & 73	Unk-7<br>ITS T SOL<br>ITS T SAG<br>16S MAMMAL<br>Soil 81 & 82	Unk-11<br>ITS T SOL<br>ITS T SAG<br>16S MAMMAL<br>Soil 89 & 90	Unk-15<br>ITS T SOL<br>ITS T SAG<br>16S MAMMAL<br>STOOL POOL 49	Unk-19<br>ITS T SOL<br>ITS T SAG<br>16S MAMMAL<br>Stool POOL 53	Unk-23<br>ITS T SOL<br><br>16S MAMMAL<br>Stool sample249					

Plate Display

	1	2	3	4	5	6	7	8	9	10	11	12
C		Unk-4<br>ITS T SOL<br>ITS T SAG<br>16S<br>MAMMAL<br>Soil 76 & 77	Unk-8<br>ITS T SOL<br>ITS T SAG<br>16S<br>MAMMAL<br>Soil 83 & 84	Unk-12<br>ITS T SOL<br>ITS T SAG<br>16S<br>MAMMAL<br>Soil 91 & 92	Unk-16<br>ITS T SOL<br>ITS T SAG<br>16S<br>MAMMAL<br>STOOL POOL 50	Unk-20<br>ITS T SOL<br>ITS T SAG<br>16S<br>MAMMAL<br>Stool POOL 54	Unk-24<br>ITS T SOL<br>ITS T SAG<br>16S<br>MAMMAL<br>Stool sample251					
D		Unk-4<br>ITS T SOL<br>ITS T SAG<br>16S<br>MAMMAL<br>Soil 76 & 77	Unk-8<br>ITS T SOL<br>ITS T SAG<br>16S<br>MAMMAL<br>Soil 83 & 84	Unk-12<br>ITS T SOL<br>ITS T SAG<br>16S<br>MAMMAL<br>Soil 91 & 92	Unk-16<br>ITS T SOL<br>ITS T SAG<br>16S<br>MAMMAL<br>STOOL POOL 50	Unk-20<br>ITS T SOL<br>ITS T SAG<br>16S<br>MAMMAL<br>Stool POOL 54	Unk-24<br>ITS T SOL<br>ITS T SAG<br>16S<br>MAMMAL<br>Stool sample251					
E		Unk-5<br>ITS T SOL<br>ITS T SAG<br>16S<br>MAMMAL<br>Soil 76 & 77	Unk-9<br>ITS T SOL<br>ITS T SAG<br>16S<br>MAMMAL<br>Soil 85 & 86	Unk-13<br>ITS T SOL<br>ITS T SAG<br>16S<br>MAMMAL<br>Soil 93	Unk-17<br>ITS T SOL<br>ITS T SAG<br>16S<br>MAMMAL<br>Stool POOL 51	Unk-21<br>ITS T SOL<br>ITS T SAG<br>16S<br>MAMMAL<br>Stool POOL 55	Unk-25<br>ITS T SOL<br>ITS T SAG<br>16S<br>MAMMAL<br>Stool sample259					
F		Unk-5<br>ITS T SOL<br>ITS T SAG<br>16S<br>MAMMAL<br>Soil 76 & 77	Unk-9<br>ITS T SOL<br>ITS T SAG<br>16S<br>MAMMAL<br>Soil 85 & 86	Unk-13<br>ITS T SOL<br>ITS T SAG<br>16S<br>MAMMAL<br>Soil 93	Unk-17<br>ITS T SOL<br>ITS T SAG<br>16S<br>MAMMAL<br>Stool POOL 51	Unk-21<br>ITS T SOL<br>ITS T SAG<br>16S<br>MAMMAL<br>Stool POOL 55	Unk-25<br>ITS T SOL<br>ITS T SAG<br>16S<br>MAMMAL<br>Stool sample259					
G	Pos-2<br>ITS T SOL<br>ITS T SAG<br>16S<br>MAMMAL<br>POS CONT T sol	Unk-6<br>ITS T SOL<br>ITS T SAG<br>16S<br>MAMMAL<br>Soil 78 & 79	Unk-10<br>ITS T SOL<br>ITS T SAG<br>16S<br>MAMMAL<br>Soil 87 & 88	Unk-14<br>ITS T SOL<br>ITS T SAG<br>16S<br>MAMMAL<br>Soil 80	Unk-18<br>ITS T SOL<br>ITS T SAG<br>16S<br>MAMMAL<br>Stool POOL 52	Unk-22<br>ITS T SOL<br>ITS T SAG<br>16S<br>MAMMAL<br>Stool POOL 56	Unk-26<br>ITS T SOL<br>ITS T SAG<br>16S<br>MAMMAL<br>Stool sample264					
H	Pos-2<br>ITS T SOL<br>ITS T SAG<br>16S<br>MAMMAL<br>POS CONT T sol	Unk-6<br>ITS T SOL<br>ITS T SAG<br>16S<br>MAMMAL<br>Soil 78 & 79	Unk-10<br>ITS T SOL<br>ITS T SAG<br>16S<br>MAMMAL<br>Soil 87 & 88	Unk-14<br>ITS T SOL<br>ITS T SAG<br>16S<br>MAMMAL<br>Soil 80	Unk-18<br>ITS T SOL<br>ITS T SAG<br>16S<br>MAMMAL<br>Stool POOL 52	Unk-22<br>ITS T SOL<br>ITS T SAG<br>16S<br>MAMMAL<br>Stool POOL 56	Unk-26<br>ITS T SOL<br>ITS T SAG<br>16S<br>MAMMAL<br>Stool sample264					

Quantification

Step #: 3
Analysis Mode: Fluorophore
Baseline Setting: Baseline Subtracted Curve Fit
Cq Determination: Single Threshold
Baseline Method:
HEX: Auto Calculated
Cy5: Auto Calculated
FAM: Auto Calculated
Threshold Setting:
HEX: 20.43, User Defined
Cy5: 13.14, User Defined
FAM: 20.49, User Defined

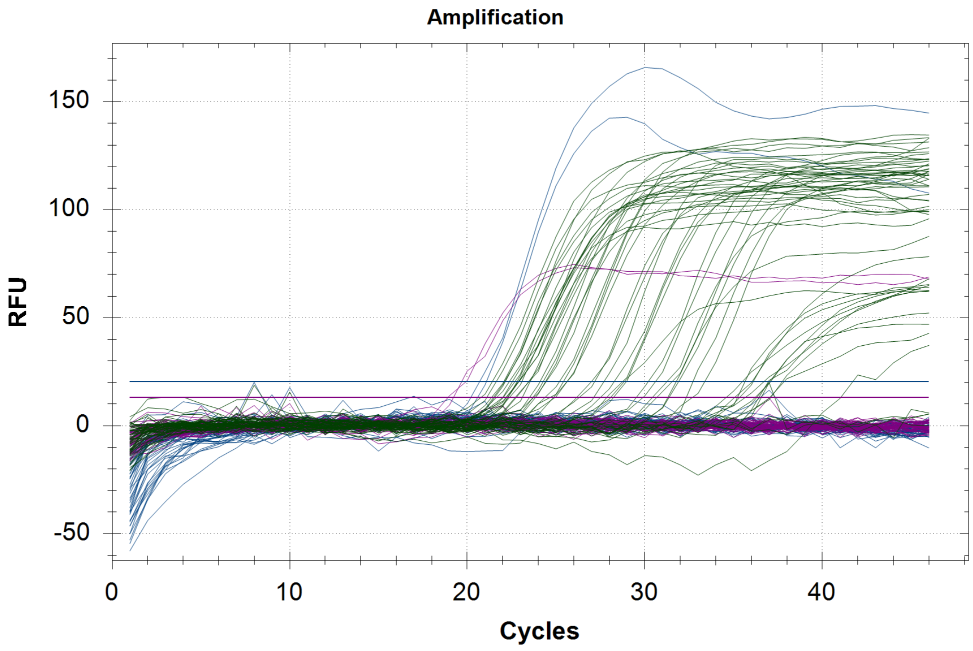

Quantification Data

Well	Fluor	Target	Content	Sample	Cq	Cq Mean	Cq Std. Dev
A01	Cy5	ITS TSAG	Pos Ctrl-01	POS CONT T sag	19,23	19,07	0,216
A02	Cy5	ITS T SAG	Unkn-03	Soil 72 & 73	N/A	0	0
A03	Cy5	ITS T SAG	Unkn-07	Soil 81 & 82	N/A	0	0
A04	Cy5	ITS T SAG	Unkn-11	Soil 89 & 90	N/A	0	0
A05	Cy5	ITS T SAG	Unkn-15	STOOL POOL 49	N/A	0	0
A06	Cy5	ITS T SAG	Unkn-19	Stool POOL 53	N/A	0	0
A10	Cy5	ITS T SAG	NTC	NTC CLEAN ROOM	N/A	0	0
A11	Cy5	ITS T SAG	NTC	NTC ADN ROOM	N/A	0	0
B01	Cy5	ITS TSAG	Pos Ctrl-01	POS CONT T sag	18,92	19,07	0,216
B02	Cy5	ITS T SAG	Unkn-03	Soil 72 & 73	N/A	0	0
B03	Cy5	ITS T SAG	Unkn-07	Soil 81 & 82	N/A	0	0
B04	Cy5	ITS T SAG	Unkn-11	Soil 89 & 90	N/A	0	0
B05	Cy5	ITS T SAG	Unkn-15	STOOL POOL 49	N/A	0	0
B06	Cy5	ITS T SAG	Unkn-19	Stool POOL 53	N/A	0	0
C02	Cy5	ITS T SAG	Unkn-04	Soil 76 & 77	N/A	0	0

Quantification Data

Well	Fluor	Target	Content	Sample	Cq	Cq Mean	Cq Std. Dev
C03	Cy5	ITS T SAG	Unkn-08	Soil 83 & 84	N/A	0	0
C04	Cy5	ITS T SAG	Unkn-12	Soil 91 & 92	N/A	0	0
C05	Cy5	ITS T SAG	Unkn-16	STOOL POOL 50	N/A	0	0
C06	Cy5	ITS T SAG	Unkn-20	Stool POOL 54	N/A	0	0
C07	Cy5	ITS T SAG	Unkn-24	Stool sample251	N/A	0	0
D02	Cy5	ITS T SAG	Unkn-04	Soil 76 & 77	N/A	0	0
D03	Cy5	ITS T SAG	Unkn-08	Soil 83 & 84	N/A	0	0
D04	Cy5	ITS T SAG	Unkn-12	Soil 91 & 92	N/A	0	0
D05	Cy5	ITS T SAG	Unkn-16	STOOL POOL 50	N/A	0	0
D06	Cy5	ITS T SAG	Unkn-20	Stool POOL 54	N/A	0	0
D07	Cy5	ITS T SAG	Unkn-24	Stool sample251	N/A	0	0
E02	Cy5	ITS T SAG	Unkn-05	Soil 76 & 77	N/A	0	0
E03	Cy5	ITS T SAG	Unkn-09	Soil 85 & 86	N/A	0	0
E04	Cy5	ITS T SAG	Unkn-13	Soil 93	N/A	0	0
E05	Cy5	ITS T SAG	Unkn-17	Stool POOL 51	N/A	0	0
E06	Cy5	ITS T SAG	Unkn-21	Stool POOL 55	N/A	0	0
E07	Cy5	ITS T SAG	Unkn-25	Stool sample259	N/A	0	0
F02	Cy5	ITS T SAG	Unkn-05	Soil 76 & 77	N/A	0	0
F03	Cy5	ITS T SAG	Unkn-09	Soil 85 & 86	N/A	0	0
F04	Cy5	ITS T SAG	Unkn-13	Soil 93	N/A	0	0
F05	Cy5	ITS T SAG	Unkn-17	Stool POOL 51	N/A	0	0
F06	Cy5	ITS T SAG	Unkn-21	Stool POOL 55	N/A	0	0
F07	Cy5	ITS T SAG	Unkn-25	Stool sample259	N/A	0	0
G01	Cy5	ITS T SAG	Pos Ctrl-02	POS CONT T sol	N/A	0	0
G02	Cy5	ITS T SAG	Unkn-06	Soil 78 & 79	N/A	0	0
G03	Cy5	ITS T SAG	Unkn-10	Soil 87 & 88	N/A	0	0
G04	Cy5	ITS T SAG	Unkn-14	Soil 80	N/A	0	0
G05	Cy5	ITS T SAG	Unkn-18	Stool POOL 52	N/A	0	0
G06	Cy5	ITS T SAG	Unkn-22	Stool POOL 56	N/A	0	0

Quantification Data

Well	Fluor	Target	Content	Sample	Cq	Cq Mean	Cq Std. Dev
G07	Cy5	ITS T SAG	Unkn-26	Stool sample264	N/A	0	0
H01	Cy5	ITS T SAG	Pos Ctrl-02	POS CONT T sol	N/A	0	0
H02	Cy5	ITS T SAG	Unkn-06	Soil 78 & 79	N/A	0	0
H03	Cy5	ITS T SAG	Unkn-10	Soil 87 & 88	N/A	0	0
H04	Cy5	ITS T SAG	Unkn-14	Soil 80	N/A	0	0
H05	Cy5	ITS T SAG	Unkn-18	Stool POOL 52	N/A	0	0
H06	Cy5	ITS T SAG	Unkn-22	Stool POOL 56	N/A	0	0
H07	Cy5	ITS T SAG	Unkn-26	Stool sample264	N/A	0	0
A02	FAM	ITS T SOL	Unkn-03	Soil 72 & 73	N/A	0	0
A03	FAM	ITS T SOL	Unkn-07	Soil 81 & 82	N/A	0	0
A04	FAM	ITS T SOL	Unkn-11	Soil 89 & 90	N/A	0	0
A05	FAM	ITS T SOL	Unkn-15	STOOL POOL 49	N/A	0	0
A06	FAM	ITS T SOL	Unkn-19	Stool POOL 53	N/A	0	0
A07	FAM	ITS T SOL	Unkn-23	Stool sample249	N/A	0	0
A10	FAM	ITS T SOL	NTC	NTC CLEAN ROOM	N/A	0	0
A11	FAM	ITS T SOL	NTC	NTC ADN ROOM	N/A	0	0
B02	FAM	ITS T SOL	Unkn-03	Soil 72 & 73	N/A	0	0
B03	FAM	ITS T SOL	Unkn-07	Soil 81 & 82	N/A	0	0
B04	FAM	ITS T SOL	Unkn-11	Soil 89 & 90	N/A	0	0
B05	FAM	ITS T SOL	Unkn-15	STOOL POOL 49	N/A	0	0
B06	FAM	ITS T SOL	Unkn-19	Stool POOL 53	N/A	0	0
B07	FAM	ITS T SOL	Unkn-23	Stool sample249	N/A	0	0
C02	FAM	ITS T SOL	Unkn-04	Soil 76 & 77	N/A	0	0
C03	FAM	ITS T SOL	Unkn-08	Soil 83 & 84	N/A	0	0
C04	FAM	ITS T SOL	Unkn-12	Soil 91 & 92	N/A	0	0
C05	FAM	ITS T SOL	Unkn-16	STOOL POOL 50	N/A	0	0
C06	FAM	ITS T SOL	Unkn-20	Stool POOL 54	N/A	0	0
C07	FAM	ITS T SOL	Unkn-24	Stool sample251	N/A	0	0

Quantification Data

Well	Fluor	Target	Content	Sample	Cq	Cq Mean	Cq Std. Dev
D02	FAM	ITS T SOL	Unkn-04	Soil 76 & 77	N/A	0	0
D03	FAM	ITS T SOL	Unkn-08	Soil 83 & 84	N/A	0	0
D04	FAM	ITS T SOL	Unkn-12	Soil 91 & 92	N/A	0	0
D05	FAM	ITS T SOL	Unkn-16	STOOL POOL 50	N/A	0	0
D06	FAM	ITS T SOL	Unkn-20	Stool POOL 54	N/A	0	0
D07	FAM	ITS T SOL	Unkn-24	Stool sample251	N/A	0	0
E02	FAM	ITS T SOL	Unkn-05	Soil 76 & 77	N/A	0	0
E03	FAM	ITS T SOL	Unkn-09	Soil 85 & 86	N/A	0	0
E04	FAM	ITS T SOL	Unkn-13	Soil 93	N/A	0	0
E05	FAM	ITS T SOL	Unkn-17	Stool POOL 51	N/A	0	0
E06	FAM	ITS T SOL	Unkn-21	Stool POOL 55	N/A	0	0
E07	FAM	ITS T SOL	Unkn-25	Stool sample259	N/A	0	0
F02	FAM	ITS T SOL	Unkn-05	Soil 76 & 77	N/A	0	0
F03	FAM	ITS T SOL	Unkn-09	Soil 85 & 86	N/A	0	0
F04	FAM	ITS T SOL	Unkn-13	Soil 93	N/A	0	0
F05	FAM	ITS T SOL	Unkn-17	Stool POOL 51	N/A	0	0
F06	FAM	ITS T SOL	Unkn-21	Stool POOL 55	N/A	0	0
F07	FAM	ITS T SOL	Unkn-25	Stool sample259	N/A	0	0
G01	FAM	ITS T SOL	Pos Ctrl-02	POS CONT T sol	21,08	20,89	0,269
G02	FAM	ITS T SOL	Unkn-06	Soil 78 & 79	N/A	0	0
G03	FAM	ITS T SOL	Unkn-10	Soil 87 & 88	N/A	0	0
G04	FAM	ITS T SOL	Unkn-14	Soil 80	N/A	0	0
G05	FAM	ITS T SOL	Unkn-18	Stool POOL 52	N/A	0	0
G06	FAM	ITS T SOL	Unkn-22	Stool POOL 56	N/A	0	0
G07	FAM	ITS T SOL	Unkn-26	Stool sample264	N/A	0	0
H01	FAM	ITS T SOL	Pos Ctrl-02	POS CONT T sol	20,7	20,89	0,269
H02	FAM	ITS T SOL	Unkn-06	Soil 78 & 79	N/A	0	0
H03	FAM	ITS T SOL	Unkn-10	Soil 87 & 88	N/A	0	0
H04	FAM	ITS T SOL	Unkn-14	Soil 80	N/A	0	0

Quantification Data

Well	Fluor	Target	Content	Sample	Cq	Cq Mean	Cq Std. Dev
H05	FAM	ITS T SOL	Unkn-18	Stool POOL 52	N/A	0	0
H06	FAM	ITS T SOL	Unkn-22	Stool POOL 56	N/A	0	0
H07	FAM	ITS T SOL	Unkn-26	Stool sample264	N/A	0	0
A01	HEX	16S MAMMAL	Pos Ctrl-01	POS CONT T sag	31,9	31,72	0,257
A02	HEX	16S MAMMAL	Unkn-03	Soil 72 & 73	35,64	35,84	0,281
A03	HEX	16S MAMMAL	Unkn-07	Soil 81 & 82	35,49	36,1	0,859
A04	HEX	16S MAMMAL	Unkn-11	Soil 89 & 90	22,87	22,97	0,144
A05	HEX	16S MAMMAL	Unkn-15	STOOL POOL 49	37,08	36,6	0,691
A06	HEX	16S MAMMAL	Unkn-19	Stool POOL 53	22,06	22,21	0,221
A07	HEX	16S MAMMAL	Unkn-23	Stool sample249	22,91	23,18	0,385
A10	HEX	16S MAMMAL	NTC	NTC CLEAN ROOM	N/A	0	0
A11	HEX	16S MAMMAL	NTC	NTC ADN ROOM	N/A	0	0
B01	HEX	16S MAMMAL	Pos Ctrl-01	POS CONT T sag	31,54	31,72	0,257
B02	HEX	16S MAMMAL	Unkn-03	Soil 72 & 73	36,04	35,84	0,281
B03	HEX	16S MAMMAL	Unkn-07	Soil 81 & 82	36,71	36,1	0,859
B04	HEX	16S MAMMAL	Unkn-11	Soil 89 & 90	23,07	22,97	0,144
B05	HEX	16S MAMMAL	Unkn-15	STOOL POOL 49	36,11	36,6	0,691
B06	HEX	16S MAMMAL	Unkn-19	Stool POOL 53	22,37	22,21	0,221
B07	HEX	16S MAMMAL	Unkn-23	Stool sample249	23,46	23,18	0,385
C02	HEX	16S MAMMAL	Unkn-04	Soil 76 & 77	37,74	39,73	2,808
C03	HEX	16S MAMMAL	Unkn-08	Soil 83 & 84	37,85	37,85	0
C04	HEX	16S MAMMAL	Unkn-12	Soil 91 & 92	22,1	22,12	0,031
C05	HEX	16S MAMMAL	Unkn-16	STOOL POOL 50	28,96	28,62	0,486
C06	HEX	16S MAMMAL	Unkn-20	Stool POOL 54	21,63	21,52	0,158
C07	HEX	16S MAMMAL	Unkn-24	Stool sample251	24,45	24,43	0,026
D02	HEX	16S MAMMAL	Unkn-04	Soil 76 & 77	41,72	39,73	2,808
D03	HEX	16S MAMMAL	Unkn-08	Soil 83 & 84	N/A	0	0

Quantification Data

Well	Fluor	Target	Content	Sample	Cq	Cq Mean	Cq Std. Dev
D04	HEX	16S MAMMAL	Unkn-12	Soil 91 & 92	22,14	22,12	0,031
D05	HEX	16S MAMMAL	Unkn-16	STOOL POOL 50	28,27	28,62	0,486
D06	HEX	16S MAMMAL	Unkn-20	Stool POOL 54	21,41	21,52	0,158
D07	HEX	16S MAMMAL	Unkn-24	Stool sample251	24,41	24,43	0,026
E02	HEX	16S MAMMAL	Unkn-05	Soil 76 & 77	28,75	28,22	0,742
E03	HEX	16S MAMMAL	Unkn-09	Soil 85 & 86	25,19	25,41	0,305
E04	HEX	16S MAMMAL	Unkn-13	Soil 93	23,54	23,51	0,04
E05	HEX	16S MAMMAL	Unkn-17	Stool POOL 51	30,6	30,4	0,288
E06	HEX	16S MAMMAL	Unkn-21	Stool POOL 55	24,1	24,19	0,126
E07	HEX	16S MAMMAL	Unkn-25	Stool sample259	25,42	25,52	0,146
F02	HEX	16S MAMMAL	Unkn-05	Soil 76 & 77	27,7	28,22	0,742
F03	HEX	16S MAMMAL	Unkn-09	Soil 85 & 86	25,62	25,41	0,305
F04	HEX	16S MAMMAL	Unkn-13	Soil 93	23,48	23,51	0,04
F05	HEX	16S MAMMAL	Unkn-17	Stool POOL 51	30,19	30,4	0,288
F06	HEX	16S MAMMAL	Unkn-21	Stool POOL 55	24,28	24,19	0,126
F07	HEX	16S MAMMAL	Unkn-25	Stool sample259	25,62	25,52	0,146
G01	HEX	16S MAMMAL	Pos Ctrl-02	POS CONT T sol	28,84	28,84	0
G02	HEX	16S MAMMAL	Unkn-06	Soil 78 & 79	33,38	33,04	0,469
G03	HEX	16S MAMMAL	Unkn-10	Soil 87 & 88	36,96	36,99	0,048
G04	HEX	16S MAMMAL	Unkn-14	Soil 80	22,9	22,72	0,256
G05	HEX	16S MAMMAL	Unkn-18	Stool POOL 52	31,25	31,51	0,371
G06	HEX	16S MAMMAL	Unkn-22	Stool POOL 56	22,81	22,74	0,089
G07	HEX	16S MAMMAL	Unkn-26	Stool sample264	27,18	27,09	0,125
H01	HEX	16S MAMMAL	Pos Ctrl-02	POS CONT T sol	N/A	0	0
H02	HEX	16S MAMMAL	Unkn-06	Soil 78 & 79	32,71	33,04	0,469
H03	HEX	16S MAMMAL	Unkn-10	Soil 87 & 88	37,03	36,99	0,048
H04	HEX	16S MAMMAL	Unkn-14	Soil 80	22,54	22,72	0,256
H05	HEX	16S MAMMAL	Unkn-18	Stool POOL 52	31,77	31,51	0,371

Quantification Data

Well	Fluor	Target	Content	Sample	Cq	Cq Mean	Cq Std. Dev
H06	HEX	16S MAMMAL	Unkn-22	Stool POOL 56	22,68	22,74	0,089
H07	HEX	16S MAMMAL	Unkn-26	Stool sample264	27	27,09	0,125

Bar Chart

Normalized expression analysis is not possible, either because no target is assigned as a reference or the selected target(s) is not appropriate. Open the Experiment Settings and assign an appropriate target as reference.

Target Names

Name	Full Name	Reference	Auto Efficiency	Efficiency
16S MAMMAL	16S MAMMAL	False	Yes	100.0%
ITS T SAG	ITS T SAG	False	Yes	100.0%
ITS T SOL	ITS T SOL	False	Yes	100.0%
ITS TSAG	ITS TSAG	False	Yes	100.0%

Sample Names

Name	Full Name	Control
POS CONT T sag	POS CONT T sag	No
POS CONT T sol	POS CONT T sol	No
Soil 83 & 84	Soil 83 & 84	No
Soil 93	Soil 93	No
Soil 72 & 73	Soil 72 & 73	No
Soil 76 & 77	Soil 76 & 77	No
Soil 78 & 79	Soil 78 & 79	No
Soil 80	Soil 80	No
Soil 81 & 82	Soil 81 & 82	No
Soil 85 & 86	Soil 85 & 86	No
Soil 87 & 88	Soil 87 & 88	No
Soil 89 & 90	Soil 89 & 90	No
Soil 91 & 92	Soil 91 & 92	No
STOOL POOL 49	STOOL POOL 49	No
STOOL POOL 50	STOOL POOL 50	No
Stool POOL 51	Stool POOL 51	No

Sample Names

Name	Full Name	Control
Stool POOL 52	Stool POOL 52	No
Stool POOL 53	Stool POOL 53	No
Stool POOL 54	Stool POOL 54	No
Stool POOL 55	Stool POOL 55	No
Stool POOL 56	Stool POOL 56	No
Stool sample249	Stool sample249	No
Stool sample251	Stool sample251	No
Stool sample259	Stool sample259	No
Stool sample264	Stool sample264	No

Gene Expression - Bar Chart Data

Target	Sample	Control	Expression	Expression SEM	Corrected Expression SEM	Mean Cq	Cq SEM	P-Value
16S MAMMAL	POS CONT T sag		N/A	N/A	N/A	31,72	0,18177	N/A
16S MAMMAL	POS CONT T sol		N/A	N/A	N/A	28,84	0	N/A
16S MAMMAL	Soil 83 & 84		N/A	N/A	N/A	37,85	0	N/A
16S MAMMAL	Soil 93		N/A	N/A	N/A	23,51	0,02802	N/A
16S MAMMAL	Soil 72 & 73		N/A	N/A	N/A	35,84	0,19895	N/A
16S MAMMAL	Soil 76 & 77		N/A	N/A	N/A	33,98	3,42587	N/A
16S MAMMAL	Soil 78 & 79		N/A	N/A	N/A	33,04	0,33138	N/A
16S MAMMAL	Soil 80		N/A	N/A	N/A	22,72	0,18114	N/A
16S MAMMAL	Soil 81 & 82		N/A	N/A	N/A	36,1	0,60722	N/A
16S MAMMAL	Soil 85 & 86		N/A	N/A	N/A	25,41	0,21578	N/A
16S MAMMAL	Soil 87 & 88		N/A	N/A	N/A	36,99	0,0342	N/A
16S MAMMAL	Soil 89 & 90		N/A	N/A	N/A	22,97	0,10161	N/A
16S MAMMAL	Soil 91 & 92		N/A	N/A	N/A	22,12	0,02171	N/A
16S MAMMAL	STOOL POOL 49		N/A	N/A	N/A	36,6	0,48876	N/A
16S MAMMAL	STOOL POOL 50		N/A	N/A	N/A	28,62	0,34361	N/A
16S MAMMAL	Stool POOL 51		N/A	N/A	N/A	30,4	0,20386	N/A

Gene Expression - Bar Chart Data

Target	Sample	Control	Expression	Expression SEM	Corrected Expression SEM	Mean Cq	Cq SEM	P-Value
16S MAMMAL	Stool POOL 52		N/A	N/A	N/A	31,51	0,26198	N/A
16S MAMMAL	Stool POOL 53		N/A	N/A	N/A	22,21	0,15605	N/A
16S MAMMAL	Stool POOL 54		N/A	N/A	N/A	21,52	0,11146	N/A
16S MAMMAL	Stool POOL 55		N/A	N/A	N/A	24,19	0,0894	N/A
16S MAMMAL	Stool POOL 56		N/A	N/A	N/A	22,74	0,06277	N/A
16S MAMMAL	Stool sample249		N/A	N/A	N/A	23,18	0,27232	N/A
16S MAMMAL	Stool sample251		N/A	N/A	N/A	24,43	0,01834	N/A
16S MAMMAL	Stool sample259		N/A	N/A	N/A	25,52	0,10292	N/A
16S MAMMAL	Stool sample264		N/A	N/A	N/A	27,09	0,08863	N/A
ITS T SAG	POS CONT T sol		N/A	N/A	N/A	N/A	N/A	N/A
ITS T SAG	Soil 83 & 84		N/A	N/A	N/A	N/A	N/A	N/A
ITS T SAG	Soil 93		N/A	N/A	N/A	N/A	N/A	N/A
ITS T SAG	Soil 72 & 73		N/A	N/A	N/A	N/A	N/A	N/A
ITS T SAG	Soil 76 & 77		N/A	N/A	N/A	N/A	N/A	N/A
ITS T SAG	Soil 78 & 79		N/A	N/A	N/A	N/A	N/A	N/A
ITS T SAG	Soil 80		N/A	N/A	N/A	N/A	N/A	N/A
ITS T SAG	Soil 81 & 82		N/A	N/A	N/A	N/A	N/A	N/A
ITS T SAG	Soil 85 & 86		N/A	N/A	N/A	N/A	N/A	N/A
ITS T SAG	Soil 87 & 88		N/A	N/A	N/A	N/A	N/A	N/A
ITS T SAG	Soil 89 & 90		N/A	N/A	N/A	N/A	N/A	N/A
ITS T SAG	Soil 91 & 92		N/A	N/A	N/A	N/A	N/A	N/A
ITS T SAG	STOOL POOL 49		N/A	N/A	N/A	N/A	N/A	N/A
ITS T SAG	STOOL POOL 50		N/A	N/A	N/A	N/A	N/A	N/A
ITS T SAG	Stool POOL 51		N/A	N/A	N/A	N/A	N/A	N/A
ITS T SAG	Stool POOL 52		N/A	N/A	N/A	N/A	N/A	N/A
ITS T SAG	Stool POOL 53		N/A	N/A	N/A	N/A	N/A	N/A
ITS T SAG	Stool POOL 54		N/A	N/A	N/A	N/A	N/A	N/A
ITS T SAG	Stool POOL 55		N/A	N/A	N/A	N/A	N/A	N/A
ITS T SAG	Stool POOL 56		N/A	N/A	N/A	N/A	N/A	N/A

Gene Expression - Bar Chart Data

Target	Sample	Control	Expression	Expression SEM	Corrected Expression SEM	Mean Cq	Cq SEM	P-Value
ITS T SAG	Stool sample251		N/A	N/A	N/A	N/A	N/A	N/A
ITS T SAG	Stool sample259		N/A	N/A	N/A	N/A	N/A	N/A
ITS T SAG	Stool sample264		N/A	N/A	N/A	N/A	N/A	N/A
ITS T SOL	POS CONT T sol		N/A	N/A	N/A	20,89	0,19032	N/A
ITS T SOL	Soil 83 & 84		N/A	N/A	N/A	N/A	N/A	N/A
ITS T SOL	Soil 93		N/A	N/A	N/A	N/A	N/A	N/A
ITS T SOL	Soil 72 & 73		N/A	N/A	N/A	N/A	N/A	N/A
ITS T SOL	Soil 76 & 77		N/A	N/A	N/A	N/A	N/A	N/A
ITS T SOL	Soil 78 & 79		N/A	N/A	N/A	N/A	N/A	N/A
ITS T SOL	Soil 80		N/A	N/A	N/A	N/A	N/A	N/A
ITS T SOL	Soil 81 & 82		N/A	N/A	N/A	N/A	N/A	N/A
ITS T SOL	Soil 85 & 86		N/A	N/A	N/A	N/A	N/A	N/A
ITS T SOL	Soil 87 & 88		N/A	N/A	N/A	N/A	N/A	N/A
ITS T SOL	Soil 89 & 90		N/A	N/A	N/A	N/A	N/A	N/A
ITS T SOL	Soil 91 & 92		N/A	N/A	N/A	N/A	N/A	N/A
ITS T SOL	STOOL POOL 49		N/A	N/A	N/A	N/A	N/A	N/A
ITS T SOL	STOOL POOL 50		N/A	N/A	N/A	N/A	N/A	N/A
ITS T SOL	Stool POOL 51		N/A	N/A	N/A	N/A	N/A	N/A
ITS T SOL	Stool POOL 52		N/A	N/A	N/A	N/A	N/A	N/A
ITS T SOL	Stool POOL 53		N/A	N/A	N/A	N/A	N/A	N/A
ITS T SOL	Stool POOL 54		N/A	N/A	N/A	N/A	N/A	N/A
ITS T SOL	Stool POOL 55		N/A	N/A	N/A	N/A	N/A	N/A
ITS T SOL	Stool POOL 56		N/A	N/A	N/A	N/A	N/A	N/A
ITS T SOL	Stool sample249		N/A	N/A	N/A	N/A	N/A	N/A
ITS T SOL	Stool sample251		N/A	N/A	N/A	N/A	N/A	N/A
ITS T SOL	Stool sample259		N/A	N/A	N/A	N/A	N/A	N/A
ITS T SOL	Stool sample264		N/A	N/A	N/A	N/A	N/A	N/A
ITS TSAG	POS CONT T sag		N/A	N/A	N/A	19,07	0,15297	N/A

QC Parameters

Data

Description	Value	Use	Results	Exclude Wells	All excluded wells
Negative control with a Cq less than	38	True		False	
NTC with a Cq less than	38	True		False	
NRT with a Cq less than	38	True		False	
Positive control with a Cq greater than	30	True	Cy5:G1, H1. HEX:A1, B1, H1.	False	
Unknown without a Cq	N/A	True	Cy5:A2, A3, A4, A5, A6, B2, B3, B4, B5, B6, C2, C3, C4, C5, C6, C7, D2, D3, D4, D5, D6, D7, E2, E3, E4, E5, E6, E7, F2, F3, F4, F5, F6, F7, G2, G3, G4, G5, G6, G7, H2, H3, H4, H5, H6, H7. FAM:A2, A3, A4, A5, A6, A7, B2, B3, B4, B5, B6, B7, C2, C3, C4, C5,	False	
Standard without a Cq	N/A	True		False	
Efficiency greater than	110.0	True			
Efficiency less than	90.0	True			
Std Curve R^2 less than	0.980	True			

Data

Description	Value	Use	Results	Exclude Wells	All excluded wells
Replicate group Cq Std Dev greater than	0.20	True	Cy5:A1, B1. FAM:G1, H1. HEX:A1, A2, A3, A5, A6, A7, B1, B2, B3, B5, B6, B7, C2, C5, D2, D5, E2, E3, E5, F2, F3, F5, G2, G4, G5, H2, H4, H5.	False	
