# Supplementary figures and images for: Baseline Assessment of Taeniasis and Cysticercosis Infections in a High-Priority Region for Taenia solium Control in Colombia
Source: Pathogens. 2025 Jul 31;14(8):755. doi: 10.3390/pathogens14080755 (PMC12388895; doi:10.3390/pathogens14080755)

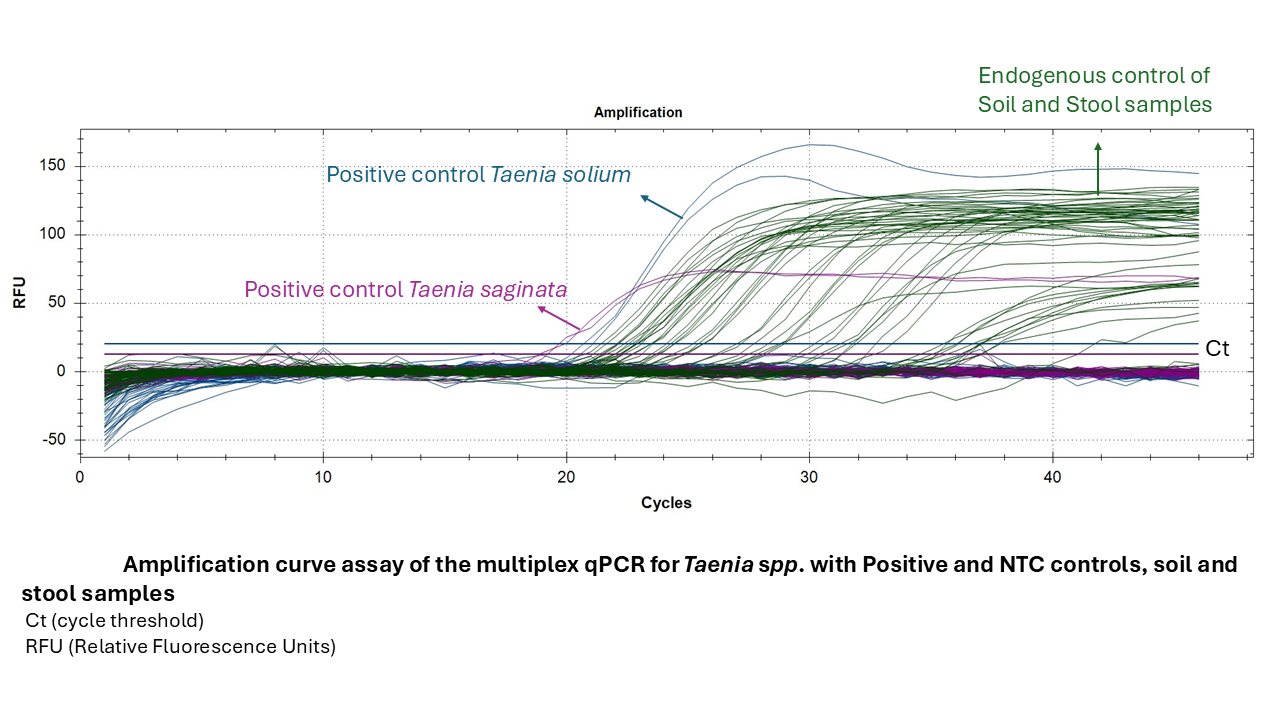

Supplement: Supplementary file 1 [file pathogens-14-00755-s001.zip › Supplementary Figure S1. Real time PCR result.tif]
